# PERSONALITY TRAITS AND VERBAL FLUENCY IN 10 COHORTS

**DOI:** 10.1093/geroni/igz038.2859

**Published:** 2019-11-08

**Authors:** Antonio Terracciano, Angelina R Sutin

**Affiliations:** Florida State University, Tallahassee, Florida, United States

## Abstract

Personality traits are associated with cognitive outcomes across the lifespan, including cognitive function in young adulthood and risk of cognitive impairment and dementia in old age. This study examined the association between the Five Factor Model personality traits and verbal fluency in 10 cohorts (11 samples) that totaled more than 90,000 participants (age range 16-101). Meta-analysis indicated that participants who scored lower in Neuroticism, and higher in Extraversion, Openness, and Conscientiousness retrieved more words, independent of age, gender, and education. These associations were consistent across semantic and letter fluency tasks. Moderation analysis indicated that the associations between personality and semantic fluency were stronger in older samples (except for Openness) and among individuals with lower education. This pattern suggests that these associations are stronger in groups vulnerable to cognitive impairment and dementia. Personality traits have pervasive associations with fluency tasks that are replicable across samples and age groups.

